# The impact of psychological distress on quality of care and access to mental health services in cancer survivors

**DOI:** 10.3389/frhs.2023.1111677

**Published:** 2023-06-19

**Authors:** Ola Abdelhadi

**Affiliations:** Division of Health Policy and Management, School of Public Health, University of California, Berkeley, CA, United States

**Keywords:** psychological distress, quality of care, cancer survivors, mental health services, access to care

## Abstract

**Introduction:**

Psychological distress is highly prevalent among cancer survivors and significantly impacts their health outcomes. Our study aim is to examine the impact of psychological distress on the quality of care in cancer survivors.

**Methods:**

We utilized longitudinal panels from the Medical Expenditure Panel Survey data spanning from 2016 to 2019 to estimate the impact of psychological distress on quality of care. We compared a sample of cancer survivors with psychological distress (*N* = 176) to a matched sample of cancer survivors without psychological distress (*N* = 2,814). We employed multivariable logistic regression models and Poisson regression models. In all models, we adjusted for age at the survey, sex, race/ethnicity, education, income, insurance, exercise, chronic conditions, body mass index, and smoking status. Descriptive statistics and regression models were performed using STATA software.

**Results:**

Our findings revealed a higher prevalence of psychological distress among younger survivors, females, individuals with lower incomes, and those with public insurance. Cancer survivors with psychological distress reported more adverse patient experiences compared to those without distress. Specifically, survivors with distress had lower odds of receiving clear explanations of their care (OR: 0.40; 95% CI: 0.17–0.99) and lower odds of feeling respected in expressing their concerns (OR: 0.42; 95% CI: 0.18–0.99) by their healthcare providers. Furthermore, psychological distress was associated with increased healthcare utilization, as evidenced by a higher number of visits (*p* = 0.02). It also correlated with a decrease in healthcare service ratings (*p* = 0.01) and the affordability of mental health services (*p* < 0.01) for cancer survivors.

**Discussion:**

These findings indicate that psychological distress can significantly impact the delivery of healthcare and the patient experience among cancer survivors. Our study underscores the importance of recognizing and addressing the mental health needs of cancer survivors. It provides insights for healthcare professionals and policymakers to better understand and cater to the mental health needs of this population.

## Introduction

Psychological distress is highly prevalent among cancer survivors and significantly affects their health outcomes and healthcare utilization, resulting in increased expenses (1). Psychological distress refers to the emotional suffering experience as a result of various psychological disorders (2). Approximately 25% of cancer survivors experience psychological distress, which can manifest as depression, anxiety, panic attacks, posttraumatic stress disorder, cancer worry, or anger (3–5). Furthermore, psychological distress can persist for up to 20 years following a cancer diagnosis, negatively impacting survivors’ health status and quality of life (6, 7). Despite the high prevalence of psychological distress among cancer survivors, little is known about how psychological distress impacts patients’ quality of care. A better understanding of how psychological distress impacts the quality of care can inform cancer care guidelines and policies to effectively manage psychological distress in clinical settings.

Psychological distress has been linked to a range of negative outcomes, including reduced quality of life, unhealthy behaviors, increased healthcare utilization, decreased treatment adherence, and higher mortality rates (8, 9). The effect of psychological distress on health outcomes is often linked to the perceived quality of care received by patients. Psychological distress can negatively affect the patient experience and satisfaction, which can further lead to overuse of healthcare services (10). Given the significant impact of psychological distress on cancer survivors’ health outcomes and healthcare service utilization, it is essential to understand how psychological distress impacts patient experiences, particularly with respect to access to mental health services. Access to mental health services is critical for effective treatment plans, but clinicians may overlook signs of psychological distress during follow-up visits, particularly when there are other pressing physical health issues and limited time for clinic visits. This oversight can exacerbate the impact of psychological distress on patients’ perception of their physical and mental health. Recognizing the importance of addressing psychological distress for high-quality cancer care, the American College Surgeons Commission on Cancer Care mandated psychological distress screening for accreditation of cancer centers in 2015 (11). This mandate underscores the need to prioritize the mental health needs of cancer survivors and ensure that they receive the necessary support to cope with the emotional toll of cancer. However, more research is needed to understand and improve the quality of mental health care services to cancer survivors, with a particular emphasis on patient experience measures (12).

Patient experience measures are widely used to evaluate the quality of care and physician performance (13). In the National Quality Strategy, patient experience is recognized as one of three primary goals, alongside improving population health and reducing healthcare costs. The Consumer Assessment of Healthcare Providers and Systems (CAHPS) is a validated tool used to measure patient experience in cancer care (14). However, cancer survivors have reported lower quality scores for patient experience compared to non-cancer adults (15). Multiple factors can affect cancer patients’ reported experiences, including social and psychological factors, as well as patient characteristics. Patient experience is not solely determined by the quality of care provided. Other factors such as psychological and social factors, as well as patients’ characteristics, can also influence their reported experiences (16). Previous studies have found that patient experience can be associated with a patient’s race, socioeconomic status, and gender (16). Additionally, sociodemographic characteristics, as well as the type and stage of cancer diagnosis, can also influence the extent of psychological distress experienced by survivors (17). For instance, survivors from ethnic minority groups may face additional cultural and social barriers to accessing mental health services. Moreover, physical health and functional impairments resulting from cancer treatment may further exacerbate psychological distress (2). Thus, it is essential to consider the unique needs of each patient when addressing psychological distress among cancer survivors.

Existing research has demonstrated a clear link between psychological factors and patients’ perceptions of care quality and treatment outcomes. In spine surgery patients, those experiencing psychological distress reported lower levels of satisfaction than their non-distressed counterparts (18). Similarly, in the context of fertility clinics, patients with lower levels of anxiety reported more positive experiences and perceived higher levels of patient-centered care than those with higher anxiety levels (19). For cancer patients, the levels of psychological distress and predictors for such distress can vary by the survivorship period (20). Health-related quality of life, particularly regarding the empathy displayed by healthcare providers, has been shown to be associated with satisfaction reported by childhood cancer survivors (21). Furthermore, perceived lower care quality has been linked to experiences of stress or depression among hematological cancer survivors (22).

There is a scarcity of research that specifically examines the quality of care provided in mental health services as its primary focus. Previous studies in the mental health field have primarily concentrated on treatment effectiveness or mortality rates (23–25), with limited information available about patient experience. Moreover, there is a deficiency of specific and validated measures to assess the quality of care in mental healthcare compared to physical healthcare. Consequently, further research is needed to develop a more comprehensive understanding of patient experience within mental health services.

This study aims to examine the relationship between psychological distress and patient experiences among cancer survivors. Our hypothesis is that higher levels of psychological distress will be associated with lower reported measures of patient experience and limited access to care among cancer survivors. Understanding the impact of psychological distress on patient experiences can aid in developing patient-centered approaches to improve the quality of care received by cancer survivors.

## Materials and methods

### Data and sample

We utilized data from the Medical Expenditure Panel Survey (MEPS) for panels 21, 22, and 23 spanning the years 2016–2019 (26). The data we used was collected through the household component of the survey, which obtained information from a representative sample of non-institutionalized individuals in the United States. This sample was selected from individuals who participated in the National Health Interview Survey. For each panel, participants were interviewed in person and also provided self-administered questionnaires over a period of two years, amounting to five rounds of data collection. The MEPS dataset includes comprehensive information on sociodemographic characteristics, health status, medical conditions, and the quality of care received (27).

We initially identified participants who self-reported a diagnosis of cancer, excluding nonmelanoma skin cancers, which aligns with previous studies that did not classify nonmelanoma skin cancer as cancer survivors (28). From our dataset, we identified a total of 3,413 individuals who were cancer survivors. Among them, 423 participants had missing information about psychological distress, and they were excluded from the analysis. This left us with a final sample of 2,990 eligible cancer survivors, with 176 reporting psychological distress and 2,814 not reporting psychological distress.

To address any potential bias from missing information about the quality of care, we conducted a sensitivity analysis. The analysis compared the percentage of psychological distress between those with missing information and those without missing information. The results of our analysis indicated that there were no significant differences in the percentage of psychological distress between the group of participants with missing information about the quality of care and the group without missing information (*p* = 0.76).

For our matched analysis, we employed propensity score matching methods to create a matched sample of cancer survivors without psychological distress. The matching was based on age, sex (male or female), and race/ethnicity (Hispanic or non-Hispanic White, Black, Asian, or other). Propensity score matching involved predicting the conditional probability of having psychological distress based on the matched covariates. Using the propensity score, we matched each cancer survivor with psychological distress to a cancer survivor without psychological distress using the nearest neighbor matching process in STATA.

## Variables

### Outcome variables

We assessed the quality of care outcomes using a self-administered questionnaire adapted from the Consumer Assessment of Healthcare Providers and Systems Clinician and Groups (CAHPS-CG) survey. This survey is widely used to measure patient experience among both cancer survivors and non-cancer patients. All the measures we used pertained to experiences within the last 12 months.

In our analysis, we focused on eight specific measures. One measure examined access to mental health services and asked whether participants “Ever delay, forgo or make changes to mental health services because of cost?”. The remaining five measures assessed various aspects of patient experience, including: (1) how often healthcare providers explained things in a way that was easy to understand, (2) how often healthcare providers showed respect for what participants had to say, (3) how often healthcare providers spent enough time with participants, (4) how often healthcare providers listened carefully to participants, and (5) how often participants received care as soon as they needed it. Participants provided responses on a 4-point Likert scale, with 1 representing “never,” 2 representing “sometimes,” 3 representing “usually,” and 4 representing “always.” To facilitate analysis, we transformed these responses into binary variables, with “never/sometimes” grouped together and “usually/always” grouped together.

Two additional measures focused on the participant’s rating of healthcare services on a scale from 0 to 10, where 0 represented the worst possible healthcare and 10 represented the best possible healthcare. Lastly, we considered utilization, which referred to the number of times a person sought care from a doctor’s office or clinic.

### Psychological distress

Psychological distress was assessed using the Kessler (K6) questionnaire, which has been widely used and validated as a screening tool for clinically significant psychological distress (29). This questionnaire has demonstrated consistency in measuring distress across various socio demographic populations.

The K6 questionnaire consisted of several questions that inquired about the frequency of certain experiences within the past 30 days. These experiences included questions about how often patients felt so sad that nothing could cheer them up; felt nervous; restless, or fidgety; felt hopeless; felt that everything was an effort, or felt worthless in the past 30 days. The response options were as follows: “none of the time” = 0, “a little of the time” = 1, “some of the time” = 2, “most of the time” = 3, and “all of the time” = 4. A total symptom score ranging from 0 to 24 was calculated based on the participant’s responses. Previous studies have established a cutoff point of 13 or higher to indicate clinically significant distress, using methods validated by prior research. Therefore, individuals with a score of 13 or above were classified as experiencing clinically significant psychological distress.

Psychological distress was assessed using the Kessler (K6) questionnaire, which has demonstrated consistency in measuring distress across various sociodemographic populations and has been validated as a screening tool for clinically significant psychological distress. The questionnaire consisted of items asking about the frequency of experiencing feelings such as sadness, nervousness, restlessness, hopelessness, lack of motivation, and worthlessness over the past 30 days. Response options ranged from “none of the time” to “all of the time” and were assigned values from 0 to 4, respectively. A total symptom score was calculated, with a score of 13 or higher indicating clinically significant distress based on established criteria used in previous studies.

### Covariates

The sociodemographic characteristics considered in this study included age, sex, education level, race/ethnicity, and family income. Family income was categorized using poverty statistics from the Current Population Survey (CPS). The income categories were defined as follows: poor (<100% of the poverty level), near poor (100% to <125% of the poverty level), low income (125% to <200% of the poverty level), middle income (200% to <400% of the poverty level), and high income (>400% of the poverty level).

Participants’ health insurance status was categorized based on their self-reported type of insurance coverage, which included private insurance, public insurance, or being uninsured. An elevated body mass index (BMI) was defined as a BMI value exceeding 25 kg/m^2^. Adverse health behaviors were assessed by participants’ current smoking status and regular exercise habits (not meeting the guideline of 150 min per week).

Chronic conditions were identified based on participants’ self-reported diagnoses of certain medical conditions. These conditions included high blood pressure, heart disease, stroke, high cholesterol, diabetes, asthma, chronic bronchitis, and arthritis.

### Analysis

Descriptive statistics, including chi-square tests and t-tests, were employed to compare the characteristics of cancer survivors with psychological distress and those without psychological distress. To estimate the impact of psychological distress and quality of care, multivariable logistic regression models were utilized. For outcomes related to the number of visits and doctors’ ratings, Poisson regression models were employed. In all models, adjustments were made for various factors, including age at the survey, sex, race/ethnicity, education, income, insurance, exercise, chronic conditions, body mass index, and smoking status. Both matched and unmatched analyses were conducted, and odds ratios were reported as the measure of effect. The descriptive statistics and regression models were performed using STATA software.

## Results

### Characteristics of cancer survivors with and without psychological distress

We conducted a comparison between two groups of cancer survivors: those with psychological distress (*N* = 176) and those without psychological distress (*N* = 2,814). Among the cancer survivors, we observed that higher levels of psychological distress were more prevalent among younger survivors, females, individuals with lower incomes, and those with public insurance as opposed to private insurance. Additionally, smoking and physical inactivity were more commonly reported among survivors with psychological distress when compared to those without psychological distress (Table 1). Moreover, there was a significant positive association between the presence of chronic conditions and psychological distress. The included cancer types in our study were listed in Table 2.

**Table 1:** Characteristics of cancer survivors with psychological distress and control group: medical expenditure panel survey 2016−2019.

Characteristics	Cancer survivors with psychological distress	Cancer survivors without psychological distress	*P*-value
	*N* = 176 (5.89%)	*N* = 2,814 (94.11%)	
Age Mean (SD)	57.65 (15.42)	65.15 (13.85)	<0.001
Sex *N* (%)			
Female	119 (6.91)	1,603 (93.09)	0.006
Male	57 (4.50)	1,211 (95.50)	
Race/Ethnicity *N* (%)			
Hispanic	27 (9.57)	255 (90.43)	0.07
Non-Hispanic-White	123 (5.32)	2,191 (94.68)	
Non-Hispanic- Black	18 (6.64)	253 (93.36)	
Non-Hispanic-Asian	<5 (5.88)	48 (94.12)	
Non-Hispanic-Other	5 (6.94)	67 (93.06)	
Poverty *N* (%)			
Poor	56 (15.82)	298 (84.18)	<0.01
Near poor	14 (10.14)	124 (89.86)	
Low income	10 (10.18)	353 (89.82)	
Middle Income	38 (5.07)	711 (94.93)	
High Income	28 (2.06)	1,328 (97.94)	
Insurance *N* (%)			
Private	64 (3.57)	1,729 (96.43)	<0.01
Public	108 (9.42)	1,039 (90.58)	
Uninsured	<5 (8.00)	46 (92.00)	
Smoking			
No	75 (4.74)	1,508 (95.26)	<0.01
Yes	39 (15.54)	212 (84.46)	
Regular Physical activity			
No	127 (8.10)	1,441 (91.90)	<0.01
Yes	39 (2.84)	1,335 (97.16)	
Having chronic conditions			
No	6 (1.70)	347 (98.3)	<0.01
Yes	170 (6.45)	2,467 (93.55)	
Explain			
Never/Sometimes	18 (18.56)	79 (81.44)	<0.01
Usually/Always	74 (4.92)	1,431 (95.08)	
Listen			
Never/Sometimes	20 (17.86)	92 (82.14)	<0.01
Usually/Always	72 (4.82)	1,421 (95.18)	
Respect			
Never/Sometimes	17 (18.28)	76 (81.72)	<0.01
Usually/Always	52 (5.05)	978 (94.95)	
Spent time			
Never/Sometimes	19 (12.67)	131 (87.33)	<0.01
Usually/Always	73 (5.01)	1,384 (94.99)	
Access to care right			
Away	15 (17.24)	72 (82.76)	<0.01
Never/Sometimes	49 (7.81)	578 (92.19)	
Usually/Always			
Doctor rating mean (SE)	7.5 (0.23)	8.5 (0.04)	<0.01

SD, standard deviation; *N*, number; SE, standard error.

*P* ≤ 0.05 = significant.

**Table 2:** Cancer type and visit number during last 12 months for cancer survivors with psychological distress and control group: medical expenditure panel survey 2016−2019.

Characteristics	Cancer survivors with psychological distress%	Cancer survivors without psychological distress%	*P*-value
Cancer types			
Melanoma	18.34	21.67	0.08
Cancer breast	16.57	20.06	
Cancer cervix	15.43	6.56	
Cancer prostate	5.14	13.78	
Cancer colon	4.73	5.80	
Cancer bladder	4.62	9.77	
Lymphoma (non-Hodgkin)	4.00	2.68	
Lung cancer	3.43	3.26	
Cancer uterus	2.96	4.61	
Other cancers	28.57	20.44	
Number of doctors’ visits			
1	18.68	13.25	<0.01
2	10.99	16.64	
3	10.99	15.18	
4	13.19	14.38	
5–9	21.98	22.77	
10 or more	24.18	17.78	

SD,  standard deviation; *N*, number; SE, standard error.

*P* ≤ 0.05 = significant.

### Health behaviors, chronic conditions, and socioeconomic status associated with psychological distress

In a multivariable logistic regression model, cancer survivors with psychological distress were more likely than cancer survivors without psychological distress to have lower incomes (OR: 1.56; 95% CI: 1.35–1.81), exercise less regularly (OR: 2.63; 95% CI: 1.61–4.17), and smoke (OR: 2.21; 95% CI: 1.40–3.51). Having at least one of the chronic conditions was associated with higher odds of having psychological distress (OR: 3.98; 95% CI: 1.75–9.07).

### Quality of care associated with psychological distress

Cancer survivors with psychological distress reported significantly lower patient-reported quality of care. They indicated that healthcare providers were less likely to explain things in a way they understood (odds ratio OR: 0.25; 95% Confidence Interval CI: 0.13–0.47), show respect for what they had to say (OR: 0.35; 95% CI: 0.18–0.65), spend enough time with them (OR: 0.44; 95% CI: 0.24–0.48), and listen carefully to them (OR: 0.31; 95% CI: 0.17–0.57). However, when a matched sample was utilized, psychological distress remained significantly associated with providers explaining things in a way they understood (OR: 0.40; 95% CI: 0.17–0.99) and providers showing respect for survivors’ perspectives (OR: 0.42; 95% CI: 0.18–0.99) (Table 3).

**Table 3:** Regression coefficients for matched and unmatched effects of psychological distress on outcomes: medical expenditure panel survey 2016–2019.

Outcome	Unmatched sample estimate (95% CI)	*P*-value	Matched sample estimate (95% CI)	*P*-value
Afford mental health services (OR)	0.91 (0.85–0.98)	0.01	0.90 (0.83–0.97)	0.005
Doctor explained well (OR)	0.25 (0.13–0.47)	0.001	0.40 (0.17–0.99)	0.05
Doctor listened well (OR)	0.31 (0.17–0.57)	0.001	0.53 (0.23–1.24)	0.14
Doctor spent enough time (OR)	0.44 (0.24–0.48)	0.005	0.75 (0.34–1.67)	0.50
Doctor show respect (OR)	0.35 (0.18–0.65)	0.001	0.42 (0.18–0.99)	0.05
Access to care right away (OR)	0.63 (0.32–1.34)	0.17	0.51 (0.22–1.22)	0.13
Doctors rating (ln)	−0.09 (−0.15–−0.03)	0.004	−0.11 (−0.21–−0.016)	0.02
Number of visits (ln)	0.14 (0.04–0.26)	0.01	0.21 (0.05–0.37)	0.01

OR, Odds Ratio for logistic regression models.

CI, Confidence Interval.

*P* ≤ 0.05 = significant.

**Figure 1:**
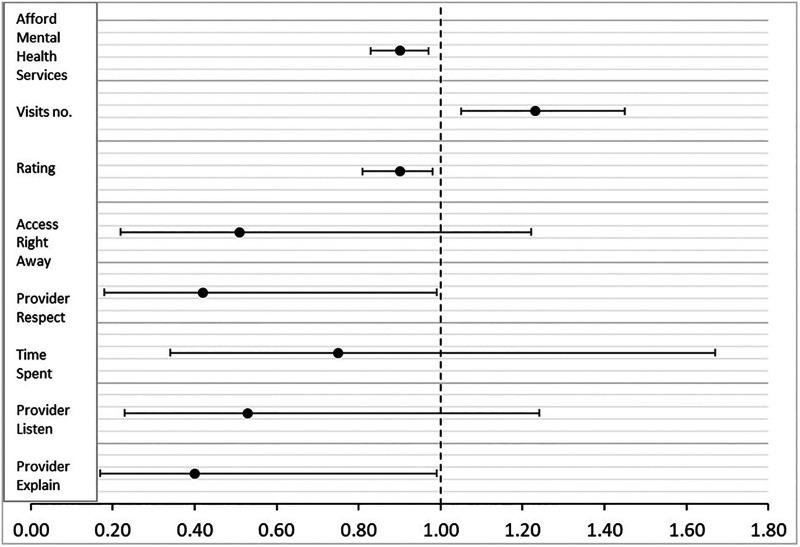
Quality and access to care associated with psychological distress: medical expenditure panel survey 2016−2019. Odds ratios and confidence intervals for quality and access to care factors associated with psychological distress. Ratios above one mean positive association and ratios below one mean negative association. The number of visits increased and health service rating, affordability of mental health services, providers respect, and explain scores decreased in patients with psychological distress compared to patients with no psychological distress.

Furthermore, health care service ratings on a scale of 0–10 were lower by 0.8 (*p* = 0.02) among cancer survivors with psychological distress compared to those without distress. Additionally, survivors with psychological distress had an increase of one additional visit every two years (*p* = 0.01) compared to survivors without psychological distress (Figure 1).

**Figure 2:**
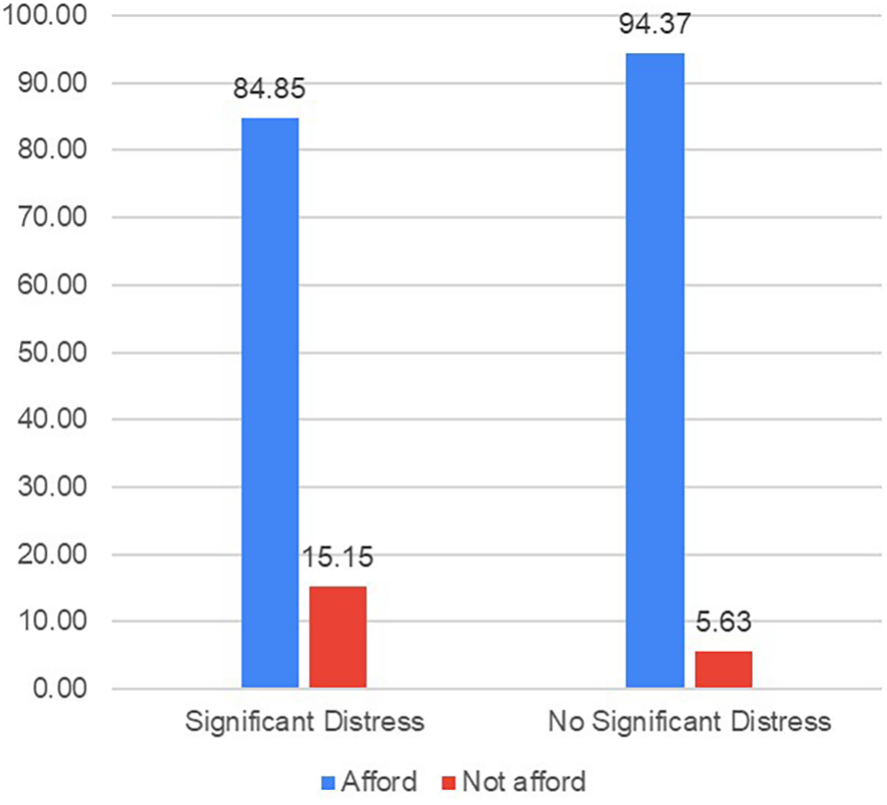
Percentage of individuals unable to afford mental health services by severity of mental distress. Rates of inability to afford mental health services among cancer survivors by psychological distress status. Survey question: “Ever delay, forgo or make changes to mental health services because of cost?” Respondents selected “yes” and “no” for the question. Kessler 6 includes a self-reported questionnaire: Participants were asked to indicate the frequency of experiencing specific feelings in the past 30 days, including feeling extremely sad to the point where nothing could cheer them up, feeling nervous, restless, or fidgety, feeling hopeless, feeling that everything was an effort, or feeling worthless. The response options ranged from “none of the time” to “all of the time” and were assigned numerical values of 0–4, respectively. A total symptom score was calculated by summing the individual item scores, resulting in a possible range of 0–24. Scores equal to or greater than 13 were indicative of clinically significant distress, based on established criteria used in prior studies.

Among cancer survivors, those with more severe psychological distress were more likely to report that mental health services were unaffordable compared to those with less severe distress (15.15% vs. 5.63%, OR: 0.90; 95% CI: 0.85–0.97, *p* < 0.01) (Figure 2).

## Discussion

Our study utilized national population-based data to examine the impact of psychological distress on various aspects of cancer survivors’ patient-reported experiences, access to mental health services, healthcare utilization, and healthcare service ratings. We found that psychological distress had a significant influence on how cancer survivors perceived their interactions with physicians, particularly in terms of understanding treatment plans and feeling respected. Furthermore, cancer survivors with psychological distress reported lower ratings for healthcare services, limited access to mental health services, and higher healthcare utilization rates. These findings align with previous research conducted on non-cancer patients and support existing literature indicating that patient satisfaction and perceived quality of care measures can be affected by the level of psychological distress (8, 18, 30–32).

Patient experience plays a crucial role in assessing the quality of care and has a significant impact on patient outcomes and healthcare costs (33). Positive patient experiences and effective communication contribute to improved care and treatment adherence, particularly for cancer survivors who often face multiple mental and physical health challenges (4). Research suggests that patient experiences are influenced by both individual characteristics and structural factors within the healthcare system (34). For instance, sociodemographic disparities can affect the patient experience, with ethnic minority patients often reporting lower satisfaction than their white patients (5). Furthermore, healthcare structural factors such as resource availability, training, and policies can also impact patient experience. In our analysis, we observed that the presence of psychological distress in patients was associated with patients reporting less time spent with healthcare providers. It is essential for providers to address not only the physical but also the mental health needs of cancer survivors. However, resource limitations, such as limited visit time and the prioritization of more urgent physical health needs, often result in the neglect of survivors’ mental health. A systematic review highlighted that primary care services for cancer patients often fail to address their psychological needs, leading to increased healthcare utilization (35).

The treatment of cancer is often complex, requiring sufficient visit time with physicians and clear explanations that patients can easily understand. Moreover, it is important to consider the psychological and cultural factors that may influence the treatment of cancer patients. Previous research has highlighted the significance of understanding patients’ culture and perceptions when assessing, diagnosing, and treating depression in this population (36). In our study, we observed that cancer patients with psychological distress reported a lack of clear explanations from their doctors in a way that they could understand. This finding aligns with previous studies that have highlighted the unmet informational needs of cancer survivors (37). Policy guidelines and healthcare institutions that prioritize patient experience as a measure of care quality can play a crucial role in supporting researchers and healthcare providers in addressing the gaps in screening and managing psychological distress among cancer survivors (38).

Psychological distress imposes a significant economic and health burden (39), particularly among cancer survivors (1, 35). Consistent with previous research, our study using data from the National Health Interview Survey (NHIS) revealed that long-term cancer survivors had a higher prevalence of psychological distress compared to adults without a history of cancer (5.6% vs. 3%) (6). In our study, we also found that 5.9% of cancer survivors reported experiencing psychological distress. Moreover, cancer survivors face challenges in accessing mental health services, primarily due to the high associated costs. Among various chronic conditions, the healthcare costs related to psychological distress are particularly high in cancer survivors (40). However, there is limited evidence on the effective management of psychosocial problems in cancer survivors within general practice settings (35). Therefore, there is a pressing need to incorporate consistent and validated screening measures for psychological distress, as well as cost-effective management protocols, into cancer survivorship care plans (41).

The higher prevalence of psychological distress among cancer survivors, coupled with the limited access to mental health services (42), underscores the importance of implementing policy interventions alongside treatment plans to address psychological distress and meet the comprehensive healthcare needs of patients (43).

Psychological distress among cancer survivors was found to be associated with lower quality of care indicators. Previous research has demonstrated that preoperative anxiety and depression in prostate cancer patients were linked to higher postoperative pain levels during hospitalization and after discharge (44). Similarly, distressed breast cancer survivors reported a greater number of treatment-related complaints (20). Depression among cancer survivors was associated with perceived lower quality of care in various aspects, including treatment delivery, treatment decision-making, follow-up care, respectful communication, patient preferences and values, and access to cancer information (22).

Conversely, good doctor-patient communication has been shown to be associated with lower psychological distress among cancer survivors (21, 45). These findings emphasize the importance of addressing psychological distress in the healthcare setting to improve the overall quality of care for cancer survivors. By focusing on effective communication and addressing the emotional needs of patients, healthcare providers can contribute to better patient experiences and outcomes in survivorship care.

Previous studies on health behavior interventions have demonstrated a significant positive impact on reducing psychological distress and improving quality of care outcomes in cancer survivors (46–48). These interventions have shown promising results in enhancing the well-being of survivors.

For instance, early implementation of cognitive-behavioral stress management programs has been found to reduce depression in breast cancer patients even up to 15 years after their diagnosis (49). This highlights the potential long-term benefits of psychological interventions in improving mental health outcomes for cancer survivors. Additionally, research has revealed a biological link between stress management and increased survival rates among cancer patients (50, 51), further emphasizing the importance of addressing psychological distress in cancer care.

Our study is subject to several limitations that should be considered when interpreting the findings. Firstly, the cross-sectional nature of the data prevents us from establishing causal relationships between psychological distress and quality of care. However, we took measures to ensure that patients reported on the quality of care after the diagnosis of psychological distress by using longitudinal panels. Secondly, the lack of information on cancer stage, severity, and time since diagnosis in the public data used for our analysis is a limitation. These factors are known to influence psychological distress in cancer survivors, and their absence may impact the generalizability of our findings. Nonetheless, previous studies have indicated that psychological distress is prevalent among cancer survivors across different cancer types and regardless of the time since diagnosis (6, 40). Thirdly, we did not have detailed information on specific psychological disorders diagnosed in the participants. Therefore, we were unable to account for the influence of specific disorders on the association between psychological distress and quality of care. However, the Kessler psychological distress survey used in our study has demonstrated high sensitivity and specificity in detecting psychological distress, as well as screening for symptoms of depression and anxiety. Finally, we were not able to study the impact of COVID-19 on psychological distress and quality of care in cancer survivors, as we were unable to include data from the COVID-19 years. The data collection process for 2020 was affected by the pandemic, leading to difficulties in pooling the data. Furthermore, the quality of care indicators, which are evaluated every other year, were last reported in 2019, and data for 2021 is currently unavailable. Finally, despite previous studies documenting the high psychological burden and unmet needs among cancer survivors during the COVID-19 pandemic (52, 53), we encountered challenges in studying the impact of COVID-19 on psychological distress and quality of care in cancer survivors, as we were unable to include data from the COVID-19 years. The data collection process for 2020 was affected by the pandemic, leading to difficulties in pooling the data. Furthermore, the quality of care indicators, which are evaluated every other year, were last reported in 2019, and data for 2021 is currently unavailable.

Future research should aim to examine the effectiveness of interventions targeting the reduction of psychological distress in improving patient experience and quality of care. By addressing these limitations and conducting further investigations, we can gain a better understanding of the complex relationship between psychological distress and quality of care in cancer survivors.

## Conclusion

High prevalence of psychological distress among cancer survivors has significant implications for the quality of care they receive. It can negatively impact patient experience, particularly when access to mental health services is limited. Providing adequate psychological support to cancer survivors is crucial for improving the quality of care, optimizing healthcare utilization, and enhancing health outcomes. This study carries important implications for healthcare providers and policymakers involved in improving the well-being of cancer survivors. These findings identify gaps in cancer survivorship care and guide the development of interventions aimed at improving access to and quality of mental health services. Furthermore, it can inform policy initiatives that promote equitable access to mental health services for cancer survivors, resulting in improved patient experiences and health outcomes.

## Data Availability

Publicly available datasets were analyzed in this study. This data can be found here: https://meps.ahrq.gov//mepsweb/data_stats/download_data_files_results.jsp?cboDataYear=All&;buttonYearandDataType=Search.
